# Nonlinearity of optoacoustic signals and a new contrast mechanism for imaging

**DOI:** 10.1038/s41377-025-01772-7

**Published:** 2025-03-27

**Authors:** Jaber Malekzadeh-Najafabadi, Jaya Prakash, Daniel Razansky, Jorge Ripoll, Vipul Gujrati, Vasilis Ntziachristos

**Affiliations:** 1 Chair of Biological Imaging, Central Institute for Translational Cancer Research (TranslaTUM), School of Medicine and Health & School of Computation, Information and Technology, Technical University of Munich, Munich, Germany; 2 Institute of Biological and Medical Imaging, Bioengineering Center, Helmholtz Zentrum München, Neuherberg, Germany; 3 Institute for Biomedical Engineering and Institute of Pharmacology and Toxicology, Faculty of Medicine, University of Zurich, Zurich, Switzerland; 4 Department of Bioengineering and Aerospace Engineering, Universidad Carlos III de Madrid, Madrid, Spain; 5 Munich Institute of Robotics and Machine Intelligence (MIRMI), Technical University of Munich, Munich, Germany; 6 Present Address: Institute for Biomedical Engineering, Department of Information Technology and Electrical Engineering, ETH Zurich, Zurich, Switzerland; 7 Present Address: Department of Instrumentation and Applied Physics, Indian Institute of Science, Bengaluru, India

**Keywords:** Photoacoustics, Nonlinear optics

## Abstract

Optoacoustic signals behave nonlinearly at light fluences above a few mJ/cm^2^, which may affect the interpretation and quantification of measurements. It has been proposed that optoacoustic nonlinearity arises from the heat-induced formation of nanobubbles or changes in local thermo-physical parameters. However, such explanations are only valid at much higher fluences than typically used in biomedical optoacoustic imaging (> 20 mJ/cm^2^) or in the presence of materials with high absorption coefficients such as gold nanoparticles. We propose herein that electromagnetic permittivity changes in response to photon absorption are major source of optoacoustic signal nonlinearity at low fluences. We provide theoretical and experimental evidence that supports this postulation and show that optoacoustic pressure responses due to permittivity changes, which are function of thermally excited third-order nonlinear susceptibility, can explain the nonlinear behavior of the optoacoustic signal. Since different materials exhibit different thermally excited third-order nonlinear susceptibility, this property could function as a new contrast mechanism that can identify the sensitivity of a substance’s dielectric constant to photon-induced temperature changes. Consequently, we propose an imaging method based on nonlinear optoacoustic signals that exploits this newly identified contrast mechanism. These findings may have far-reaching implications for improving the accuracy of optoacoustics and utilizing the proposed new contrast mechanism would advance our understanding of cellular and tissue functionality.

## Introduction

Nonlinearity has been observed for light fluences above 6 mJ/cm^2^ in both optical-resolution optoacoustic microscopy, which uses focused illumination, and in acoustic-resolution optoacoustic imaging, which uses broad-beam illumination^1–5^. This nonlinear behavior depends on the light wavelength employed, at lower wavelengths the nonlinearity is stronger, and the properties of the sample, by increasing absorption and scattering coefficient the nonlinearity is getting stronger^5^.

While previous studies have provided extensive insights into the mechanisms of optoacoustic nonlinearity, such as local temperature increases causing changes in thermo-physical parameters like the Grüneisen parameter and thermal expansion coefficient^3,4,6–9^ or saturation of the absorption coefficient^4^, as well as material phase effects like fluid evaporation or nano-bubble formation^1,2^, these explanations may not fully account for the nonlinearity observed at low fluence and low absorption coefficient. Although temperature rise may be implicated in the generation of optoacoustic nonlinearity at high illumination fluences or in the presence of gold nanoparticles, which have high plasmon-resonance-based absorption coefficients^6–9^, it is unclear whether the moderate fluences (< 20 mJ/cm^2^) employed in biomedical measurements generate nonlinearity through temperature effects when the absorption coefficient of the target is low. Indeed, the instantaneous temperature rise from pulsed light sources at moderate fluence is on the order of milli-Kelvins^10^, which is insufficient to cause nano-bubble formation^2^ or significant variations in the Grüneisen parameter^11^.

In this work, we theoretically and experimentally investigated alternate sources of optoacoustic nonlinearity at moderate light fluences (<20 mJ/cm^2^). We observed a nonlinear behavior in optoacoustic signals at low fluences and low absorption coefficients, which is particularly prominent in the frequency domain of the signal. We first investigated the source of this non-linearity in a theoretical dielectric slab by deriving the relationship between generated optoacoustic pressure and a change in permittivity, which is a function of third-order nonlinear susceptibility. We validated this relationship by observing variations in the shape of optoacoustic signals as a function of fluence in an agar phantom, which approximates a dielectric slab. These experiments indicate that thermally excited variations in the permittivity cause a nonlinear change in optoacoustic pressure with fluence. Furthermore, both theory and experiment show that these nonlinear changes are more prominent at higher optoacoustic signal frequencies. Our findings suggest that, in conditions of low fluence and low absorption, optoacoustic nonlinearity can arise from mechanisms other than those traditionally considered, thereby expanding our understanding of optoacoustic signal generation under these conditions.

Optoacoustic imaging methods typically assume that optoacoustic signal intensity is proportional to the illumination fluence^12,13^. However, not accounting for nonlinearity may lead to erroneous estimates of a target substance’s properties, such as chromophore concentrations or tissue pathophysiology. Based on our postulation herein, we developed a methodology to account for nonlinear variations of optoacoustic signals and improve the accuracy of measurements. Moreover, we propose a novel imaging methodology that exploits thermally excited third-order nonlinear susceptibility as its contrast mechanism. In tissues, this new contrast mechanism relates to the sensitivity of the dielectric constants of cells and organelles to photon-induced heat. We exploit this contrast mechanism to deliver the first images of thermally excited third-order nonlinear susceptibility, and more generally non-linearity, in phantoms and in vivo tissues.

### Theory

In this section, we theoretically investigate the contributions of the Grueneisen parameter, absorption coefficient, and permittivity to optoacoustic nonlinearity. We show that the changes in the Grueneisen parameter and absorption coefficient of a material cannot explain the optoacoustic nonlinearity observed under low light fluences (<20 mJ/cm^2^) when the absorption coefficient of the target is low. We further show that the nonlinear changes in optoacoustic pressure can be caused by thermally excited changes in the permittivity $(\Delta {\varepsilon }_{{\rm{th}}})$, which is a function of thermally excited third-order nonlinear susceptibility $({\chi }_{{\rm{th}}}^{\left(3\right)})$. Next, we derive the acoustic wave equation for optoacoustic pressure $({p}_{{\rm{th}}})$ and the change in pressure $(\Delta {p}_{{\rm{th}}})$ in order to investigate the nonlinear variations in the detected optoacoustic signal.

### Calculating nonlinear variations in optoacoustic pressure

To study the origins of optoacoustic non-linearity, we began by investigating the effects of an electric field on a theoretical absorptive dielectric slab in a parallel plate capacitor (Figure S1), which provides a simple approximation of the interaction of light with tissue^14^. Tissues, especially biological tissues, contain water and electrolytes, which give them dielectric properties. These properties allow tissue to behave similarly to a dielectric material when exposed to an electric field. As discussed in ref. ^14^ dielectric slabs in a parallel-plate capacitor model experience forces under an electric field, resulting in nonlinear optical effects. By using this model, we simplify the interaction of light with tissue. The change in the temperature of the dielectric slab induced by the generated heat per unit of volume is given by (see Supplementary Note 1 for the derivation of Eq. (1)),

1 $$\Delta T=\frac{{\mu }_{{\rm{a}}}\varphi }{\rho {C}_{p}}$$

where *φ* is the light fluence (Jm^−2^), *μ*_a_ indicates optical absorption coefficient (m^−1^), *C*_*p*_ represents specific heat capacity $({\rm{J}}.{{\rm{K}}}^{-1}.K{g}^{-1})$ and *ρ* indicates the mass density (*Kgm*^−3^). Equation (1) assumes adiabatic conditions, neglecting heat transport, which is a reasonable assumption on the timescale of photoacoustic signal generation. When the standard conditions for tissue-mimicking phantom measurements are inserted into Eq. (1) $(\varphi =10{\rm{mJc}}{{\rm{m}}}^{-2},\rho =1000{\rm{kg}}/{{\rm{m}}}^{3}{\rm{and}}{C}_{p}=4.18{\rm{J}}/({\rm{gK}})$ for water, and ${\mu }_{a}=0.1{\rm{c}}{{\rm{m}}}^{-1}$ at wavelength 800 nm; see Materials and methods section Phantoms for details), the change in temperature is ∆*T* = 0.24 mK, which is generally too small to alter thermo-physical parameters of the sample sufficiently to induce nonlinearity (see Supplementary Note 3 for effects of temperature on optoacoustic nonlinearity)^10^.

The measured optoacoustic signal is the generated initial pressure rise inside the material that is propagated and then detected by the transducer. To find the main source of the nonlinear variations in optoacoustic signals, we should obtain a general form of the pressure equation, which includes all possible nonlinear parameters. We first calculate the pressure generated by the changes in the temperature. The pressure, thermally excited by the transferred heat can be written as (see Supplementary Note 1 for the derivation of Eq. (2)),

2 $${p}_{{th}}={\Gamma \mu }_{a}\varphi$$

where Г is the Grueneisen parameter. Equation (2) therefore represents the optoacoustic pressure generated by heat. However, as we show in Supplementary Notes 2 and 3, the contributions of the Grueneisen parameter and the absorption coefficient to nonlinear variations in optoacoustic pressure are negligible at moderate fluences (<20 mJ/cm^2^) and low absorption coefficients.

Next, we investigated the variation in the optoacoustic pressure due to the change in the permittivity, which is a function of temperature and pressure^14^. The change in pressure becomes (See Supplementary Note 1 for the derivation of Eq. (3)),

3 $$\Delta {p}_{{th}}=\frac{\Delta {\varepsilon }_{{th}}}{2{nc}}I$$

where $\Delta {p}_{{th}}$ is the change in optoacoustic pressure due to the thermally excited change in the real part of the permittivity $(\Delta {\varepsilon }_{{th}})$, *I* is the field intensity $({{\rm{Js}}}^{-1}{{\rm{m}}}^{-2})$, n represents refractive index, and c indicates speed of light (m/s). Therefore, the dielectric slab (Figure S1) experiences a total pressure,

4 $${p}_{{tot}}={p}_{{th}}+\Delta {p}_{{th}}={\Gamma \mu }_{a}\varphi +\frac{\Delta {\varepsilon }_{{th}}}{2{nc}}I$$

By replacing *φ* with $\int {\rm{I}}({\rm{t}}){\rm{dt}}$, we see that $\Delta {p}_{{th}}$ is proportional to the derivative of thermal pressure,

5 $$\Delta {p}_{{th}}\propto \frac{\partial {p}_{{th}}}{\partial {\rm{t}}}$$

Equation 5 implies that the nonlinear variation in the optoacoustic pressure can be calculated by taking the derivative of the thermal pressure.

Equation (4) represents the local increase in pressure upon excitation with light. However, in order to investigate non-linear variations in detected optoacoustic pressure, we must first derive an acoustic pressure wave equation (see Supplementary Note 1 for the derivation of Eq. (6)), which is represented for a symmetric cylindrical coordinate system along the z-axis by,

6 $${\nabla }^{2}p\left(r,t\right)-\frac{1}{{v}^{2}}\frac{{\partial }^{2}}{\partial {t}^{2}}p\left(r,t\right)=-\frac{{\Gamma \mu }_{a}}{{v}^{2}}\frac{\partial }{\partial {\rm{t}}}{\rm{I}}\left(r,t\right)-\frac{\Delta {\varepsilon }_{{th}}}{2{nc}{v}^{2}}\frac{{\partial }^{2}}{\partial {{\rm{t}}}^{2}}{\rm{I}}\left(r,t\right)$$

where *v* is speed of sound (m/s). If an acoustic detector (transducer) is placed at position z < 0, the detected acoustic signal would include both thermal optoacoustic pressure and nonlinear variations in optoacoustic pressure (See Supplementary Note 1 for the derivations of Eqs. (7) and (8)):

7 $${\widetilde{{\rm{p}}}}_{{\rm{th}}}^{-}\left({\rm{z}},{\rm{\omega }}\right)={M}_{1}{I}_{0}$$

8 $$\Delta {\widetilde{p}}_{{th}}^{-}\left({\rm{z}},{\rm{\omega }}\right)={M}_{2}{I}_{0}^{2}{\rm{\omega }}$$

where, ${\widetilde{{\rm{p}}}}_{{\rm{th}}}^{-}$ and $\Delta {\widetilde{p}}_{{th}}^{-}$ are thermal pressure and the variation in optoacoustic pressure, respectively; ${M}_{1}=-\frac{{{\rm{I}}}_{{\rm{\omega }}}{{\rm{I}}}_{1}}{2v}{\Gamma \mu }_{a}\exp \left({\rm{ikz}}\right)$ and ${M}_{2}=-i\frac{{{\rm{I}}}_{{\rm{\omega }}}{{\rm{I}}}_{1}}{2v}\frac{{\chi }_{{th}}^{(3)}}{2{\varepsilon }_{0}{\left({\rm{nc}}\right)}^{2}}\exp ({\rm{ikz}})$, where ${{\rm{\chi }}}_{{th}}^{(3)}$(${m}^{2}/{V}^{2}$) is the third-order nonlinear susceptibility due to thermal effects, ${I}_{0}$ represents the initial field intensity, ${\varepsilon }_{0}$ is vacuum permittivity, ${\rm{k}}=\frac{{\rm{\omega }}}{v}$, ${{\rm{I}}}_{{\rm{\omega }}}$ is the Fourier transform of ${{\rm{I}}}_{{\rm{t}}},{{\rm{I}}}_{1}={\int }_{{Z}^{{\prime} }}^{{\infty }}{{\rm{I}}}_{{\rm{z}}}\exp \left(-{\rm{ikz}}\right){\rm{dz}}$.

In order to determine whether the nonlinear variation in optoacoustic pressure, $\Delta {\widetilde{p}}_{{th}}^{-}$, contributes to nonlinearity in the detected optoacoustic pressure, the relationships in Eqs. (4)-(8) can be tested experimentally in the frequency and time domains. Equations (7) and (8) show that ${\widetilde{{\rm{p}}}}_{{\rm{th}}}^{-}$ is a linear function of light intensity that is independent of frequency (*ω*), while $\Delta {\widetilde{p}}_{{th}}^{-}$ is a function of both light intensity and frequency. Because $\Delta {\widetilde{p}}_{{th}}^{-}$ is a nonlinear function of light intensity, the nonlinearity of the measured optoacoustic pressure should become stronger at higher frequencies. To evaluate $\Delta {\widetilde{p}}_{{th}}^{-}$ in the time domain, the calculated initial pressure rise from Eq. (6) can be written as (See supplementary Note 1 for the derivation of Eq. (9)),

9 $${p}_{{tot}}={p}_{{th}}+\Delta {p}_{{th}}={\Gamma \mu }_{a}\Delta {\rm{t}}\,{\rm{I}}+\frac{{\chi }_{{th}}^{(3)}}{2{\varepsilon }_{0}{\left({nc}\right)}^{2}}{I}^{2}$$

where ∆t is the laser pulse duration. Equation (9) shows that the total optoacoustic pressure includes both *p*_*th*_ and ∆*p*_*th*_, which behave linearly and nonlinearly with changing light energy, respectively. Since the thermal pressure (*p*_*th*_) is a linear function of light intensity, we can extract the nonlinear contribution of ∆*p*_*th*_ to the total pressure $({p}_{{tot}})$ by subtracting two normalized values of ${p}_{{tot}}$ measured at two different light intensities. By using Eq. (9), we find that

10 $$\Delta {\widetilde{p}}_{{tot}}=\frac{{p}_{{tot}2}}{{I}_{2}}-\frac{{p}_{{tot}1}}{{I}_{1}}=\frac{{\chi }_{{th}}^{\left(3\right)}}{2{\varepsilon }_{0}{\left({nc}\right)}^{2}}\Delta I$$

where ${p}_{{toti}}$ is the total optoacoustic pressure at light intensity *I*_*i*_ and $\Delta I$ is ${I}_{2}-{I}_{1}$. By comparing Eq. (10) with $\Delta {p}_{{th}}$ in Eq. (9), we find that $\Delta {\widetilde{p}}_{{tot}}$ (Eq. (10)) equals the normalized value of $\Delta {p}_{{th}}$ for a given change in light intensity $\Delta I$, yielding,

11 $$\Delta {\widetilde{p}}_{{tot}}\propto \Delta {\widetilde{p}}_{{th}}=\frac{\Delta {p}_{{th}}}{I}$$

where $\Delta {\widetilde{p}}_{{th}}$ is the normalized value of $\Delta {p}_{{th}}$.

### Development of an algorithm to reconstruct images of nonlinear variations in optoacoustic pressure

To reconstruct an image of the nonlinear variation in optoacoustic pressure, we must solve the acoustic wave equation (Eq. (6)) and extract $\Delta {\widetilde{p}}_{{th}}$ (Eq. (10) and Eq. (11)), which should enable us to develop a reconstruction algorithm to produce an image that represents the thermally excited third-order nonlinear susceptibility (${{\chi }}_{{th}}^{(3)}$ in Eq. (10)). According to the definition of ${{{\chi }}}_{{th}}^{(3)}$, a reconstructed image using the developed algorithm would represent a map of the ability of the sample to change its dielectric constant in response to photon-induced heat. Image reconstruction was then based on a Poisson-type integral, which is the analytical solution of Eq. (6),

12 $$p\left(r,t\right)={N}_{1}\frac{\partial }{\partial {\rm{t}}}\mathop{\int }\nolimits_{l(t)}\frac{{\mu }_{a}{\rm{I}}}{\left|r-r^{\prime} \right|}{dl}(t)+{N}_{2}\frac{{\partial }^{2}}{\partial {{\rm{t}}}^{2}}\mathop{\int }\nolimits_{l(t)}\frac{\left|{\chi }_{{th}}^{(3)}\right|{I}^{2}}{\left|r-r^{\prime} \right|}{dl}(t)$$

where $l(t)$ is the path in 2-D, $\left|r-r^{\prime} \right|={vt},{N}_{1}=\frac{\Gamma }{4\pi v},{N}_{2}=\frac{1}{8{\rm{\pi }}{\varepsilon }_{0}{\left({nc}\right)}^{2}v}$ and $\Delta {\varepsilon }_{{th}}$ is replaced by $\frac{{\chi }_{{th}}^{(3)}I}{n{\varepsilon }_{0}c}$. According to Eq. (10), we can rewrite Eq. (12) for collected optoacoustic signals at two different light intensities as,

13 $$\Delta {\widetilde{p}}_{{tot}}={N}_{2}\frac{{\partial }^{2}}{\partial {{\rm{t}}}^{2}}\mathop{\int }\nolimits_{l(t)}\frac{\left|{\chi }_{{th}}^{(3)}\right|\Delta {\rm{I}}}{\left|r-r^{\prime} \right|}{dl}(t)$$

where $\Delta {\rm{I}}$ is ${I}_{2}-{I}_{1}$ and light intensity is assumed to be constant. By developing a model-based reconstruction algorithm for Eq. (13), we can reconstruct images of ${\chi }_{{th}}^{(3)}$ in tissue. The value of $\Delta {\widetilde{p}}_{{tot}}$ can be obtained from two optoacoustic measurements recorded at two different light intensities by using Eq. (10). To develop a new model-based algorithm, we altered the algorithm described by Dean-Ben et al. ^12^ by rewriting Eq. (13) as,

14 $$\Delta {\widetilde{p}}_{{tot}}\approx \frac{H\left(t+\Delta t\right)-2H\left(t\right)+H\left(t-\Delta t\right)}{{\left(\Delta t\right)}^{2}}$$

where $$H\left(t\right)$$ is

15 $$H\left(t\right)=\mathop{\int }\nolimits_{l(t)}\frac{\left|{\chi }_{{th}}^{(3)}\right|\Delta {\rm{I}}}{\left|r-r^{\prime} \right|}{dl}(t)$$

Standard optoacoustic image reconstruction algorithms assume that the measured overall signal (*p*_*tot*_) is equivalent to the thermal pressure (*p*_*th*_). We quantitatively estimated that the magnitude of nonlinear changes in optoacoustic pressure (∆*p*_*th*_) is much larger than that of the Grueneisen parameter (See supplementary Note 4). However, the above theoretical findings (Eqs. (7)-(11)) prompted us to investigate whether the variations in thermal pressure (∆*p*_*th*_) may contribute significantly to a measured optoacoustic signal. If true, then ∆*p*_*th*_ could potentially be calculated from measured optoacoustic data that behaves nonlinearly. Furthermore, knowing the contribution of $\Delta {p}_{{th}}$ to the optoacoustic signal would allow us to reconstruct the images of thermally excited third-order nonlinear susceptibility $({\chi }_{{th}}^{\left(3\right)})$ as a new parameter for a given sample.

## Results

In this section, we experimentally verify the above theoretical findings, which show the existence of nonlinear variations in optoacoustic pressure due to ${\chi }_{{th}}^{(3)}$, in both the frequency domain (Eq. (8)) and the time domain (Eqs. (10) and (11)). We then apply the developed reconstruction algorithm to produce images of nonlinear variations in optoacoustic pressure in a phantom and in tissue. In section Investigating nonlinear optoacoustic variations in the frequency domain, the data collected from a tissue-mimicking phantom is analyzed in frequency domain to validate our theoretical results (Eqs. (7) and (8)) by calculating the Fourier transform of measured optoacoustic signals and exploring the nonlinear variations in the amplitude of the optoacoustic spectrum at different frequency components. The nonlinear variations in optoacoustic pressure (∆*p*_*th*_) are also investigated in the time domain in section Investigating nonlinear optoacoustic variations in the time domain by extracting $\Delta {p}_{{th}}$ (Eqs. (10) and (11)) from optoacoustic signals collected from a tissue-mimicking phantom. In section Phantom and In-vivo imaging, we reconstruct an image of a phantom that consists of two solutions (pure ethanol and distilled water) and an in-vivo dataset (the kidney cross-section of a mouse) to evaluate the performance of the developed reconstruction algorithm (Section “Development of an algorithm to reconstruct images of nonlinear variations in optoacoustic pressure”).

### Investigating nonlinear optoacoustic variations in the frequency domain

**Fig. 1:**
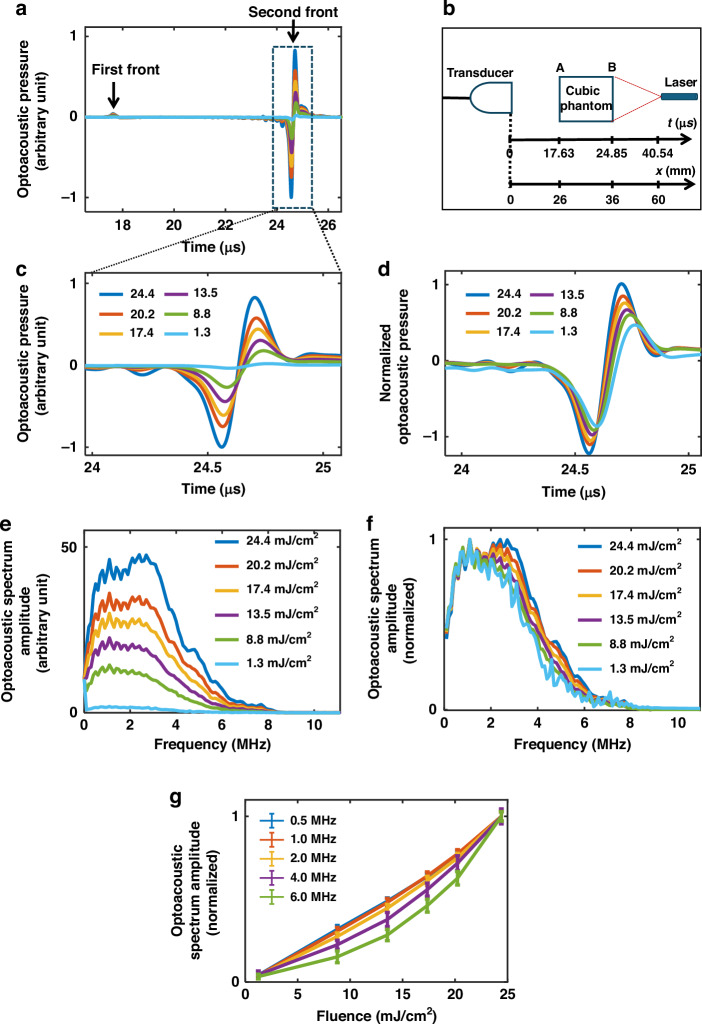
Nonlinear changes in the optoacoustic spectrum and signal as a function of fluence. A homogeneous agar cube with uniform reduced scattering coefficient ${{\rm{\mu }}}_{{\rm{s}}}^{\prime} =4{\pm 1{\rm{cm}}}^{-1}$ and absorption coefficient ${{\rm{\mu }}}_{{\rm{a}}}=0.1\pm 0.02{{\rm{cm}}}^{-1}$ was illuminated with a laser operating at 800 nm at different light fluences. **a** The raw optoacoustic signal as a function of time (depth). The signals corresponding to the edge of the cube closer to transducer is weaker than the signals from the edge closer to illumination source due to light attenuation. **b** Diagram of the location of the phantom in relation to the illumination source and transducer in terms of both time and distance. **c** Enlarged plot of the signal from the edge closest to the illumination source. **d** The same signals from panel **c** normalized to the corresponding fluences. **e** The spectra of the signals in panel **a**. **f** The signals from panel **e** normalized to the corresponding fluences. **g** The optoacoustic spectrum amplitude at different frequencies as a function of fluence

Our theoretical considerations (Eq. (8)) indicate that there may be nonlinear variations in the measured optoacoustic pressure with light intensity due to ${{\rm{\chi }}}_{{th}}^{(3)}$. In order to confirm these theoretical findings, we illuminated an optically diffusive phantom at different fluences and assessed the behavior of the detected optoacoustic signal. We examined and characterized the existence of nonlinear variations $(\Delta {\widetilde{p}}_{{th}}^{-})$ in the signal as a function of light fluence by using Eq. (8). The phantom comprising a homogeneous agar cube with a uniform absorption coefficient $({\mu }_{{\rm{a}}}=0.1\pm 0.02{{\rm{cm}}}^{-1})$ and a reduced scattering coefficient $({\mu }_{{\rm{s}}}^{{\prime} }=4\pm 1{{\rm{cm}}}^{-1})$ was illuminated with various fluences at 800 nm in transmission mode. At all fluences, we observed an initial signal corresponding to the pressure generated at the edge of the cube closest to the transducer (Fig. 1a and b, edge A), which is relatively weak due to light attenuation through the phantom, followed by a second stronger signal corresponding to the pressure generated at the edge closer to the illumination source (Fig. 1a and b, edge B). Figure 1c shows the second signal in greater detail. The optoacoustic signals were normalized to the fluences at which they were measured (Fig. 1d). We next calculated the Fourier transform of each measured optoacoustic signal at different light fluences (Fig. 1e). Figure 1f shows the normalization of panel (e). Figure 1g shows the optoacoustic spectrum amplitude as a function of fluence at different frequencies. The optoacoustic spectrum amplitude becomes more nonlinear at higher frequencies (Fig. 1g), which is consistent with an increasing contribution of $\Delta {\widetilde{p}}_{{th}}^{-}$ (Eq. (8)) to the total optoacoustic pressure at higher frequencies. Conversely, the amplitude is nearly linear at low frequencies, corresponding to the negligible contribution of $\Delta {\widetilde{p}}_{{th}}^{-}$ (Eq. (8)) to the total optoacoustic pressure at frequencies lower than 1 MHz (Fig. 1g). We can therefore interpret the observed nonlinearity (Fig. 1g) as the nonlinearity in $\Delta {\widetilde{p}}_{{th}}^{-}$. Note that the Grueneisen parameter and the absorption coefficient cannot be the cause of the observed nonlinearity (Fig. 1f and g) because the nonlinearity in these parameters are independent of acoustic frequency (See Supplementary Note 2 and 3 for nonlinear variation in optoacoustic pressure due to absorption coefficient and Grueneisen parameter, respectively). Two-photon absorption was also considered, but as discussed in the supplementary section ‘Two-Photon Absorption (TPA),’ this effect is negligible and cannot account for the observed nonlinearities (See Supplementary Note 5).

### Investigating nonlinear optoacoustic variations in the time domain

In this section, we analyze the influence of nonlinear variations in optoacoustic pressure $(\Delta {p}_{{th}})$ on the total measured optoacoustic signals in the time domain by using the theoretical description of $\Delta {p}_{{th}}$ (Eqs. (10) and (11)). Using the acquired data from the homogeneous agar cube (Fig. 1), we show that nonlinear variations in optoacoustic pressure are proportional to the derivative of the thermal pressure (Eq. (5)) and can be calculated by subtracting two normalized values of total optoacoustic pressure measured at two different light intensities (Eq. (11)).

**Fig. 2:**
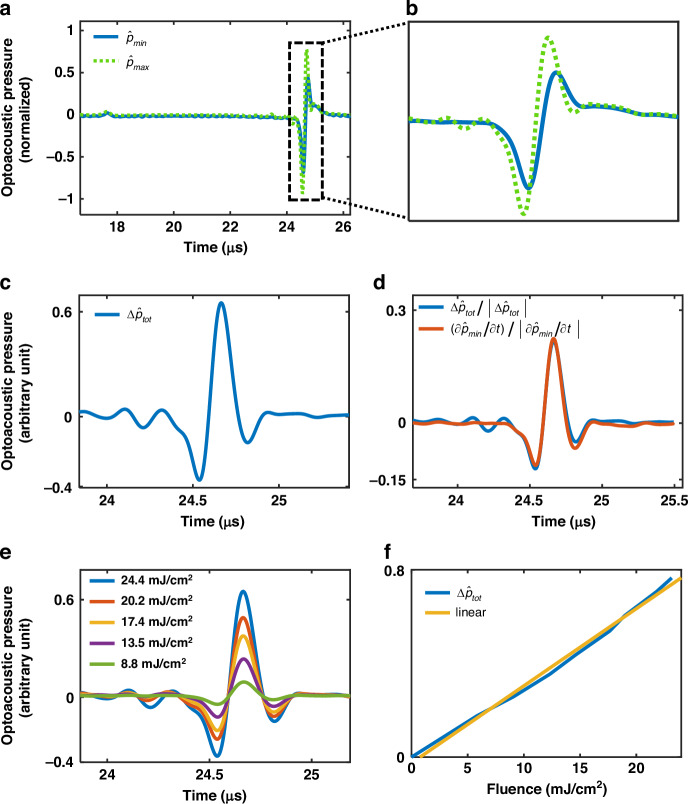
Exploring the nonlinear variations in optoacoustic pressure as a function of fluence. In panels **a**, **e**, a homogeneous agar cube with uniform absorption coefficient ${\mu }_{{\rm{a}}}=0.1\pm 0.02{{\rm{cm}}}^{-1}$ and reduced scattering coefficient ${\mu }_{{\rm{s}}}^{\prime} =4\pm 1{{\rm{cm}}}^{-1}$ was illuminated with laser operating at 800 nm with different fluence levels on the sample. **a** and **b** ${\hat{p}}_{\min }$ and ${\hat{p}}_{\max }$ are the optoacoustic signal at fluence 1.3$\pm 0.02$ and 24.4 ± 0.1 mJ/cm^2^, respectively, which are normalized to the corresponding fluence. **c** $\Delta {\widetilde{p}}_{{tot}}$ as the result of subtracting ${\hat{p}}_{\min }$ from ${\hat{p}}_{\max }$. **d** The similarity of $\Delta {\widetilde{p}}_{{tot}}$ and $\frac{\partial {p}_{\min }}{\partial {\rm{t}}}$ as $\frac{\partial {p}_{{th}}}{\partial {\rm{t}}}$ after normalization. **e** The calculated $\Delta {\widetilde{p}}_{{tot}}$ for different fluences by subtracting ${\hat{p}}_{\min }$ from the optoacoustic signals normalized by the corresponding fluences. **f** The maximum value of calculated $\Delta {\widetilde{p}}_{{tot}}$ in panel **e** over the variation of the corresponding fluence with respect to the minimum fluence (1.3 ± 0.02 mJ/cm^2^) and a linear function fitting

Figure 2a and b show the optoacoustic signals at the lowest $(1.3\pm 0.02\frac{{\rm{mJ}}}{{{\rm{cm}}}^{2}})$ and highest $(24.2\pm 0.1\frac{{\rm{mJ}}}{{{\rm{cm}}}^{2}})$ fluences, normalized to their corresponding fluence values and designated as ${\hat{p}}_{\min }=\frac{{p}_{\min }}{{I}_{\min }}{\rm{and}}\,{\hat{p}}_{\max }=\frac{{p}_{\max }}{{I}_{\max }}$. If the total optoacoustic pressure includes only the linear thermal pressure, the ${\hat{p}}_{\min }$ and ${\hat{p}}_{\max }$ should overlap; however, ${\hat{p}}_{\min }$ and ${\hat{p}}_{\max }$ differ in both phase and amplitude. We assume the observed nonlinearity is due to $\Delta {p}_{{th}}$, which can be extracted from the total measured optoacoustic pressure by using Eq. (10). By substituting *p*_min_ and *p*_max_ for ${p}_{{tot}1}$ and ${p}_{{tot}2}$, respectively, into Eq. (10), we find that

16 $$\Delta {\widetilde{p}}_{{tot}}={\hat{p}}_{\max }-{\hat{p}}_{\min }=\frac{{\chi }_{{th}}^{\left(3\right)}}{2{\varepsilon }_{0}{\left({nc}\right)}^{2}}\Delta I$$

where $\Delta I$ is ${I}_{\max }-{I}_{\min }$. By using Eqs. (11) and (16), we find that

17 $$\Delta {\widetilde{p}}_{{tot}}=\frac{\Delta {p}_{{th}}}{\Delta I}$$

where $\Delta {p}_{{th}}$ is represented by $\frac{\Delta {\varepsilon }_{{th}}}{2{nc}}\Delta I$ (Eq. (3)). Figure 2c gives $\Delta {\widetilde{p}}_{{tot}}$, which is calculated by subtracting ${\hat{p}}_{\max }$ and ${\hat{p}}_{\min }$ (Eq. (16)). To evaluate our calculations (Eqs. (16) and (17)), we can use $\Delta {p}_{{th}}\propto \frac{\partial {p}_{{th}}}{\partial {\rm{t}}}$ (Eq. (5)) and $\Delta {\widetilde{p}}_{{tot}}\propto \Delta {\widetilde{p}}_{{th}}$ (Eq. (11)), in which $\Delta {\widetilde{p}}_{{tot}}$ is proportional to the derivative of the thermal pressure,

18 $$\Delta {\widetilde{p}}_{{tot}}\propto \frac{\partial {p}_{{th}}}{\partial {\rm{t}}}$$

Since $\Delta {p}_{{th}}$ is nonlinear with respect to fluence (Eq. (9)), the contribution of $\Delta {p}_{{th}}$ to the optoacoustic signal at low fluence $({p}_{\min })$ can be neglected. Therefore, we can approximate the thermal pressure $({p}_{{th}})$ by ${\hat{p}}_{\min }$ in Eq. (18), in which case we find from Eq. (18) that,

19 $$\frac{\Delta {\widetilde{p}}_{{tot}}}{\left|\Delta {\widetilde{p}}_{{tot}}\right|}\cong \frac{\frac{\partial {p}_{\min }}{\partial {\rm{t}}}}{\left|\frac{\partial {p}_{\min }}{\partial {\rm{t}}}\right|}$$

Figure 2d shows the normalization of both $\frac{\partial {p}_{\min }}{\partial {\rm{t}}}$ and $\Delta {\widetilde{p}}_{{tot}}$, which fit each other well, validating Eq. (19) and supporting that the observed nonlinearity in total measured optoacoustic pressure is due to $\Delta {p}_{{th}}$.

Since the nonlinear variation in optoacoustic pressure (∆*p*_*th*_) is a second-degree polynomial function of light intensity (Eq. (9)), then its normalization (Eq. (17)) should be a linear function of light intensity (∆*I*, Eq. (16)). Therefore, we can confirm that $\Delta {p}_{{th}}$ behaves nonlinearly by showing that the change in total optoacoustic pressure $(\Delta {\widetilde{p}}_{{tot}})$ is a linear function of ∆*I*. To calculate $\Delta {\widetilde{p}}_{{tot}}$ at various fluences, we subtract the normalized optoacoustic signals at the lowest fluence $({\hat{p}}_{\min })$ from all other measured optoacoustic signals, which are subsequently normalized by the fluences at which they were measured (Fig. 2e). Figure 2f plots the maximum value of $\Delta {\widetilde{p}}_{{tot}}$ (see Fig. 2e) as a function of fluence, showing that the relationship is indeed linear. Therefore, we can then consider $\Delta {p}_{{th}}$ to be a second-degree polynomial function of fluence, which is consistent with theory (Eqs. (9) and (10)).

### Phantom and In-vivo imaging

We have thus far showed that nonlinear variations in optoacoustic pressure (∆*p*_*th*_) can influence the measured optoacoustic signal. The ability to record such variations could allow the use of $\Delta {p}_{{th}}$ as a new contrast mechanism relating to thermally excited third-order nonlinear susceptibility $({{\rm{\chi }}}_{{th}}^{(3)})$ of matter. Consequently, we developed an image reconstruction algorithm (Section “Development of an algorithm to reconstruct images of nonlinear variations in optoacoustic pressure) to produce images of ${{\rm{\chi }}}_{{th}}^{(3)}$ contrast by using $\Delta {p}_{{th}}$. In order to verify our assumption that the reconstructed data from the new algorithm represents ${{\rm{\chi }}}_{{th}}^{(3)}$, we recorded data from a phantom comprising two materials with known values of ${{\rm{\chi }}}_{{th}}^{(3)}$ (calculated from $\Delta {\varepsilon }_{{th}}$ using Eq. S19, see^15^) using a multispectral optoacoustic tomography scanner (MSOT, see Materials and methods). The phantom contained two plastic tubes with diameters of 250 *μm*, which contained pure ethanol and distilled water. Black ink was added to both tubes to achieve a uniform absorption coefficient of ${\mu }_{{\rm{a}}}=0.1\pm 0.02{{\rm{cm}}}^{-1}$ (see Materials and methods). Optoacoustic measurements were acquired at two light fluences ${\varphi }_{\min }=1.5\pm 0.02$ and ${\varphi }_{\max }=9\pm 0.1{\rm{mJ}}/{{\rm{cm}}}^{2}$ to extract the nonlinear variation of the optoacoustic pressure (∆*p*_*th*_), where ${\hat{p}}_{\min }=\frac{{p}_{\min }}{{\varphi }_{\min }}{\rm{and}}\,{\hat{p}}_{\max }=\frac{{p}_{\max }}{{\varphi }_{\max }}$. Note that the maximum illumination of $9\pm 0.1{\rm{mJ}}/{{\rm{cm}}}^{2}$ is typical for clinical MSOT imaging, i.e. the measurements performed did not exceed the approved fluence limits for in vivo imaging.

**Fig. 3:**
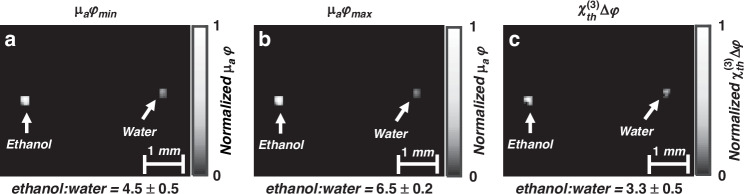
Phantom optoacoustic imaging. **a** and **b** Reconstructed image of absorption coefficient at light fluence 1.5 ± 0.02 and 9 ± 0.1 mJ/cm^2^, respectively, 200 kHz - 8 MHz band-pass filter. **c** Reconstructed image of thermally excited third-order nonlinear susceptibility $({{\rm{\chi }}}_{{th}}^{(3)})$ by using the modified model-based algorithm, according to Eqs. (14) and (15). Ratio values show the ratio between the mean value of the reconstructed data at Ethanol and Water tubes

Figure 3 shows images of the phantom, reconstructed by a standard model-based algorithm (Fig. 3a, b) and by our modified algorithm (Fig. 3c). Relative ratios of the mean pixel intensities in the two tubes (ethanol:water) were used to compare the images. Figure 3a, b show images of the phantom at two light fluences of 1.5 ± 0.02 and 9 ± 0.1 mJ/cm^2^ (800 nm), reconstructed using a standard model-based algorithm^12^, which considers ${p}_{\min }$ and ${p}_{\max }$ as purely thermal pressure (see Materials and methods). The ethanol:water pixel intensity ratios are 4.5 ± 0.5 and 6.5 ± 0.2 for the low and high fluences, respectively, demonstrating the nonlinear behavior of the optoacoustic signal. We next used the two acquired data sets (*p*_min_ and *p*_max_) to produce an image of the thermally excited third-order nonlinear susceptibility $({{\rm{\chi }}}_{{th}}^{(3)})$ contrast (Fig. 3c), which is reconstructed using the modified model-based algorithm (Section “Development of an algorithm to reconstruct images of nonlinear variations in optoacoustic pressure”); note that light intensity is proportional to fluence, therefore ∆I can be replaced by $\Delta {\rm{\varphi }}={\varphi }_{\max }-{\varphi }_{\rm{mim}}$ in Eq. (15)). The ethanol:water pixel intensity ratio of the resulting image is 3.28, which is consistent with the expected ratio of ${{\rm{\chi }}}_{{th}}^{(3)}$ for ethanol and water (3.37)^15^.

**Fig. 4:**
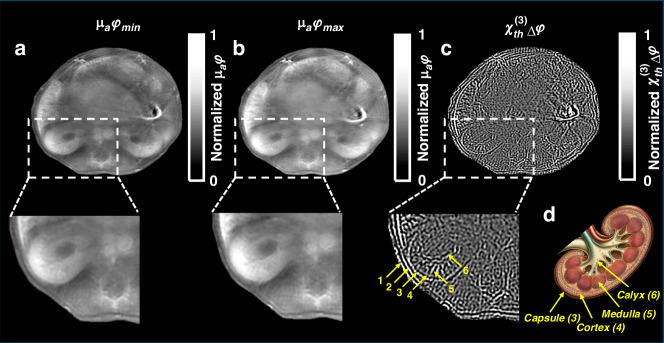
Optoacoustic imaging taken in-vivo through a mouse kidney cross-section. **a**, **b** Reconstructed image of absorption coefficient at light fluence 1.5 ± 0.02 and 9 ± 0.1 mJ/cm^2^, respectively, showing the entire optoacoustic data without further processing to extract specific structures. **c** Image of thermally excited third-order nonlinear susceptibility $({{\rm{\chi }}}_{{th}}^{(3)})$, reconstructed using the modified algorithm according to Eqs. (14) and (15). No post-processing, such as vessel extraction using filters like Frangi filter, was applied. The aim is to illustrate the contrast mechanisms rather than the detailed visualization of vessel structures. Arrows 1 and 2 show the skin and muscle of the abdomen, respectively. Arrows 3-6 indicate structures within the kidney (Arrow 3: Capsule, Arrow 4: Cortex, Arrow 5: Medulla, and Arrow 6: Calyx). **d** Anatomical reference for the kidney structures

In order to test the modified algorithm on in-vivo measurements, we studied images from the kidney of a live mouse (see Materials and methods). Optoacoustic measurements were acquired at two light fluences, ${\varphi }_{\min }=1.5\pm 0.02$ and ${\varphi }_{\max }=9\pm 0.1{\rm{mJ}}/{{\rm{cm}}}^{2}$, and labeled ${p}_{\min }$ and ${p}_{\max }$, respectively. Figure 4 shows images of the mouse kidney cross-section, reconstructed using a standard model-based algorithm (Fig. 4a-b) and the modified algorithm (Fig. 4c) in order to investigate the new contrast $({{\rm{\chi }}}_{{th}}^{(3)})$. Figure 4a and b show images of the mouse kidney at light fluences $1.5\pm 0.02\,({{\mu }_{a}\varphi }_{\min })$ and 9 ± 0.1 mJ/cm^2^ (${{\mu }_{a}\varphi }_{\max }$; 800 nm illumination), respectively, reconstructed using the linear model-based algorithm^16^. Figure 4c shows the same cross-section, reconstructed with the modified model-based algorithm (Eq. (14)) using the two acquired data sets as input (fluences of 1.5 ± 0.02 and 9 ± 0.1 mJ/cm^2^). The contrast of the reconstructed image in Fig. 4c represents the difference in the permittivities of the tissues between the two illumination fluences, which is proportional to the thermally excited third-order nonlinear susceptibility $({{\rm{\chi }}}_{{th}}^{(3)})$. The edges and fine structures in the image clearly resemble the high-frequency component of the data acquired, which is consistent with our assertion that the nonlinear changes in optoacoustic pressure are more significant at high frequencies (Eq. (8)). Arrows 1 and 2 show the skin and muscle surrounding the abdomen, respectively. Arrows 3-6 show the anatomy of kidney: Arrow 3, Capsule; Arrow 4, Cortex; Arrow 5, Medulla; and Arrow 6, Calyx^17^. Anatomical reference for the kidney structures is shown in Fig. 4d.

To verify the stability and reproducibility of the ${{\rm{\chi }}}_{{th}}^{(3)}$ measurements, we repeated the experiment three times at different fluences and analyzed the results. The corresponding data, including the average and standard deviation of the measurements across multiple regions of interest (ROIs), are provided in Supplementary Note 6. This analysis demonstrates the consistency of the reconstructed ${{\rm{\chi }}}_{{th}}^{(3)}$ images across repeated experiments.

Temperature variations during measurements were minimized due to controlled experimental conditions. Any potential temperature changes would affect a larger area due to blood circulation, impacting low frequencies that are linear and, according to our theory, do not influence the observed nonlinearities in ${{\rm{\chi }}}_{{th}}^{(3)}$. This approach ensures that the measured nonlinearities are primarily due to ${{\rm{\chi }}}_{{th}}^{(3)}$ and not artifacts of temperature fluctuation.

**Fig. 5:**
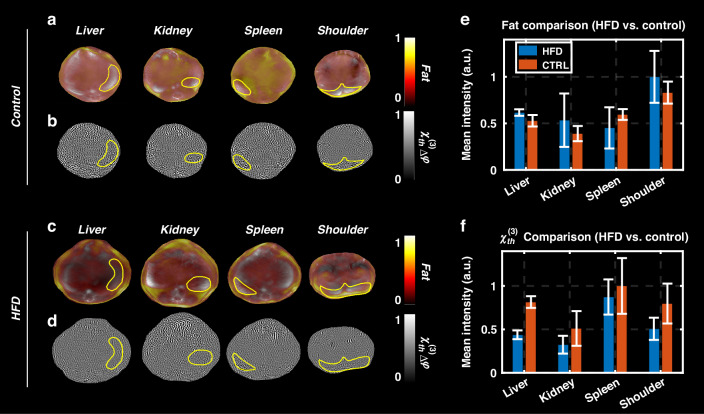
Optoacoustic and ${{\boldsymbol{\chi }}}_{{\boldsymbol{th}}}^{\left({\bf{3}}\right)}$ imaging data acquired in vivo for the fat study. **a** Unmixed fat images overlaid on optoacoustic anatomical images (background at 800 nm) for a mouse from the Control group, showing fat distribution across four cross-sections: liver, kidney, spleen, and shoulder. **b** Corresponding reconstructed ${{\boldsymbol{\chi }}}_{{\boldsymbol{th}}}^{\left({\bf{3}}\right)}$ images for the cross-sections shown in panel **a**. **c** Unmixed fat images overlaid on optoacoustic anatomical images (background at 800 nm) for a mouse from the HFD group, showing fat distribution across the same four cross-sections: liver, kidney, spleen, and shoulder. **d** Corresponding reconstructed ${{\boldsymbol{\chi }}}_{{\boldsymbol{th}}}^{\left({\bf{3}}\right)}$ images for the cross-sections shown in panel **c**. **e** Comparison of mean fat intensity in different organs between the HFD and Control (CTRL) groups (three mice per group). **f** Comparison of mean ${{\boldsymbol{\chi }}}_{{\boldsymbol{th}}}^{\left({\bf{3}}\right)}$ intensity in different organs between the HFD and Control (CTRL) groups (three mice per group). Mean intensities in panels **e** and **f** were calculated within the regions of interest (ROIs) of organs, as demonstrated for the two mice shown in panels **a**–**d**

Figure 5 presents the optoacoustic and ${\chi }_{{th}}^{\left(3\right)}$ imaging results obtained from fat (HFD) and normal (CTRL) mice. This study aimed to evaluate fat distribution and ${{\rm{\chi }}}_{{th}}^{\left(3\right)}$ values across key tissues (liver, kidney, spleen, and shoulder) and compare the two groups to explore physiological differences associated with a high-fat diet. The analysis was performed for 6 mice (3 per group).

Unmixed fat images from representative mice in the Control and HFD groups, overlaid on optoacoustic anatomical images (800 nm background), are shown in Fig. 5a and c, respectively. These images clearly visualize fat distribution patterns across the liver, kidney, spleen, and shoulder sections. Comparisons of fat intensity, averaged across regions of interest (ROIs) for each organ, are shown in Fig. 5e. ROIs for intensity calculations are illustrated in Fig. 5a–d for two example mice. The fat intensity values in Fig. 5e represent the mean intensities calculated across all mice in each group.

Reconstructed ${{\rm{\chi }}}_{{th}}^{\left(3\right)}$ images corresponding to the same cross-sections are presented in Fig. 5b and d for the Control and HFD groups, respectively. Comparisons of ${{\rm{\chi }}}_{{th}}^{\left(3\right)}$ intensity values, averaged across ROIs for each organ, are shown in Fig. 5f. Interestingly, ${{\rm{\chi }}}_{{th}}^{\left(3\right)}$ intensities were generally lower in the HFD group compared to the Control group for all organs. This observation suggests that ${{\rm{\chi }}}_{{th}}^{\left(3\right)}$ is sensitive to compositional and thermal property changes in tissues caused by a high-fat diet.

Figure 5e shows the fat intensity was higher in the HFD group compared to the Control group for most organs, consistent with the expected increase in fat deposition due to the high-fat diet, except for the spleen. The highest fat accumulation was observed in the shoulder region for the HFD group. Notably, in the spleen, ${{\rm{\chi }}}_{{th}}^{\left(3\right)}$ values in the HFD group were lower than in the Control group, consistent with the trends observed in other organs (Fig. 5f). However, panel (e) shows that fat values in the spleen were unexpectedly lower in the HFD group compared to the Control group, which contradicts expected trends. This discrepancy suggests that fat intensity measurements in the spleen might not fully capture the anticipated differences, whereas ${{\rm{\chi }}}_{{th}}^{\left(3\right)}$ provides a more reliable indication of tissue compositional changes.

The liver exhibited the most pronounced differences in ${{\rm{\chi }}}_{{th}}^{\left(3\right)}$ between the groups (Fig. 5f), reflecting its critical metabolic role and high susceptibility to fat accumulation. In contrast, fat intensity in the liver showed only a small difference between the HFD and Control groups (Fig. 5e). The spleen and kidney showed relatively smaller changes in ${{\rm{\chi }}}_{{th}}^{\left(3\right)}$ compared to the liver, which may be due to their distinct physiological responses to a high-fat diet and varying degrees of fat accumulation.

These results highlight the reliability of ${{\rm{\chi }}}_{{th}}^{\left(3\right)}$ as a contrast mechanism for detecting tissue-level differences caused by fat deposition, as it consistently aligns with expected physiological trends across organs, even when fat intensity measurements deviate. ${{\rm{\chi }}}_{{th}}^{\left(3\right)}$ therefore provides a complementary and robust tool for studying tissue compositional changes in response to a high-fat diet.

Additionally, ${{\rm{\chi }}}_{{th}}^{\left(3\right)}$ images in Fig. 5b for the Control group show clearer vascular and organ edge structures compared to Fig. 5d for the HFD group. These results, along with the ${{\rm{\chi }}}_{{th}}^{\left(3\right)}$ intensity trends in Fig. 5f, suggest that ${{\rm{\chi }}}_{{th}}^{\left(3\right)}$ can serve as a novel contrast mechanism for evaluating fat-induced changes in tissue composition. This promising potential warrants further investigation to fully explore the utility of ${{\rm{\chi }}}_{{th}}^{\left(3\right)}$ imaging in biomedical applications.

## Discussion

We propose that the origin of nonlinear variations of optoacoustic signals at fluences typical of biomedical optoacoustic imaging is primarily due to thermally excited third-order nonlinear susceptibility. This postulation deviates from previous assumptions that associated nonlinear responses with the formation of nanobubbles^1,2^, changes in thermo-physical parameters^3,10^ or saturation of the absorption coefficient^4^. The difference to previous studies is that the regime of our study relates to lower fluences, i.e. deposited energies that are not sufficient to cause these effects. Nevertheless, even at lower fluences, our results suggest that not accounting for thermally excited third-order nonlinear susceptibility $({{\rm{\chi }}}_{{th}}^{\left(3\right)})$ could lead to quantification errors, as observed in Figs. 2b and 4c. If certain tissues, dyes, or particles used as contrast agents exhibit significant ${{\rm{\chi }}}_{{th}}^{\left(3\right)}$, this could affect imaging outcomes, particularly in optoacoustic microscopy and mesoscopy where higher laser energies are used. Investigating these potential impacts systematically is our next step.

The nonlinear variations in optoacoustic pressure can be extracted from the measured data and reconstructed to generate an image (Fig. 4c), whose contrast represents the change in the ${{\rm{\chi }}}_{{th}}^{\left(3\right)}$ of the imaged structures. The non-linear signals collected are substantial and can be extracted as a new form of contrast. Using a novel algorithm, proposed herein, we deliver the first images of thermally excited third-order nonlinear susceptibility $({{\rm{\chi }}}_{{th}}^{\left(3\right)})$, essentially introducing a new imaging modality, or a new ability to optoacoustic imaging. A reconstructed image of a mouse kidney using this algorithm demonstrates that differences in permittivity between different tissues and organs yield differences in contrast (Fig. 4c). We further found that the nonlinear changes in pressure are more prominent at higher frequencies, which manifests in the visibility of finer structures in the images (Fig. 4c, consistent with Eq. (8)).

In the fat study, we investigated the potential of thermally excited third-order nonlinear susceptibility $({{\rm{\chi }}}_{{th}}^{\left(3\right)})$ as a novel contrast mechanism for optoacoustic imaging, focusing on its sensitivity to tissue compositional and thermal property changes induced by a high-fat diet. Our results demonstrated that ${{\rm{\chi }}}_{{th}}^{\left(3\right)}$ imaging provides complementary information to conventional optoacoustic imaging, offering insights beyond fat distribution alone.

Fat (HFD) and normal (CTRL) mice were compared across multiple organs (liver, kidney, spleen, and shoulder). While optoacoustic signals were influenced by tissue depth and light fluence, ${{\rm{\chi }}}_{{th}}^{\left(3\right)}$ imaging revealed distinct differences in tissue composition. Interestingly, ${{\rm{\chi }}}_{{th}}^{\left(3\right)}$ intensities were consistently lower in the HFD group, potentially reflecting tissue-specific responses to fat deposition. Additionally, ${{\rm{\chi }}}_{{th}}^{\left(3\right)}$ imaging displayed clearer structural details, such as vascular and organ edge maps, particularly in the Control group.

These findings highlight ${{\rm{\chi }}}_{{th}}^{\left(3\right)}$ as a promising biomarker for detecting diet-induced tissue changes and as a complementary imaging modality to optoacoustic signals. By providing information independent of optical absorption, ${{\rm{\chi }}}_{{th}}^{\left(3\right)}$ may enhance the sensitivity and specificity of optoacoustic imaging in applications such as metabolic studies and disease diagnostics.

${{\rm{\chi }}}_{{th}}^{\left(3\right)}$ could also vary with physiological conditions. It is expected to be sensitive to changes in tissue architecture, such as alterations in tissue density, water content, or metabolic activity—factors often associated with pathological conditions like cancer, fibrosis, or inflammation. This unique sensitivity could allow ${{\rm{\chi }}}_{{th}}^{\left(3\right)}$ imaging to provide real-time feedback on disease progression or response to treatment, enabling longitudinal monitoring of the same tissue over time^18–21^.

Further studies are necessary to fully explore the potential of ${{\rm{\chi }}}_{{th}}^{\left(3\right)}$ imaging, including its application in various physiological and pathological conditions, and its role as a new contrast mechanism for advanced biomedical imaging.

Our proposed methodology for un-mixing and reconstructing the contribution of nonlinear changes to the total measured optoacoustic pressure should be validated with more biological samples and developed as a tool for obtaining new types of functional information. Moreover, the methodology shown to capture non-linear responses could be employed to improve the accuracy and fidelity of optoacoustic tomography. Such development could potentially be extended to other optical imaging modalities, such as fluorescence molecular tomography and diffuse optical tomography. Moreover, as mentioned above, our results may also have important implications for nonlinear behavior in optoacoustic microscopy and mesoscopy.

## Materials and methods

### Phantoms

Tissue-mimicking homogeneous phantoms (analyzed in Figs. 1 and 2) were prepared as cubes (1 ×1 x 1 cm) by mixing agar (2% solution in deionized water, 05039-500 G, Sigma-Aldrich, Steinheim, Germany) with Black India ink (Higgins, Texas) and Intralipid (20% emulsion, I141-100ML, Sigma). The ink was first diluted using deionized water as needed in order to obtain the desired final absorption coefficients, as determined using a spectrometer (LS-1-cal, USB4000; Ocean Optics, Germany). The amount of Intralipid was also varied in order to achieve different reduced scattering coefficients^22^. First, the ink and Intralipid were mixed, then the 2% agar was added, and the entire mixture was heated in a microwave oven.

The data analyzed in Fig. 3 was acquired from two plastic tubes with inner diameter of 250 *μm*. The plastic tube has a negligible absorption coefficient at wavelength 800 nm. We used pure ethanol and distill water mixed by Black India ink (Higgins, Texas) in order to obtain the desired absorption coefficient.

### In vivo imaging

Nude mice (Envigo) and B6 (Cg)-Tyrc-2J/J mice (*n* = 3 per group, Jackson Lab) was used for in vivo optoacoustic images using an MSOT inVision 256-TF (iThera Medical, Munich, Germany). Animals were fed with a high-calorie diet (D12331i, Research Diets) or a normal diet. Animals were scanned under standard imaging conditions^23^. All procedures involving animal experiments were approved by the Animal Care and Handling Office of Helmholtz Zentrum München and the Government of Upper Bavaria.

### Optoacoustic data acquisition

**Fig. 6:**
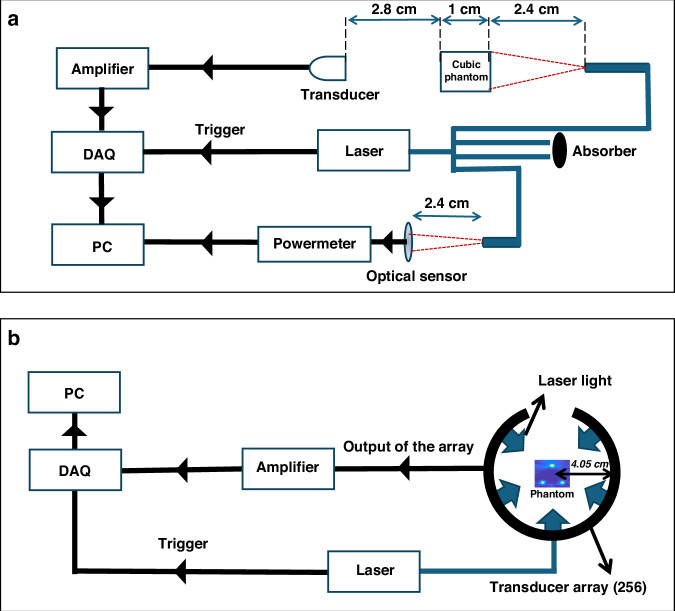
Schematic of the set-ups used to study nonlinear changes in optoacoustic pressure with phantoms and in-vivo. **a** The set-up used to collect optoacoustic signal from homogeneous diffusive phantoms, for which the experimental results are shown in Figs. 1–2. **b** The set-up used to collect optoacoustic signal from a mouse kidney cross-section, in-vivo, for which the results are shown in Figs. 3–5

To investigate the nonlinear changes in optoacoustic pressure in phantoms, the results of which are shown in Figs. 1 and 2, we used the experimental setup shown in Fig. 6a. The cubic phantom was aligned with the transducer and with the illumination source inside a chamber filled with deionized water. The cubic phantom was illuminated using a tuneable optical parametric oscillator (OPO) laser (InnoLas Laser, Germany). The cubic phantom and the optical sensor of a power meter (PE50BF-DIF-C RoHS, OPHIR, Darmstadt, Germany) were located at the same distance (2.4 cm) from the illumination source to be able to measure accurately the fluence on the surface of the phantom. To achieve the same illumination (with a Gaussian beam profile) on the surface of the optical sensor and phantom, a four-branch fiber bundle (WF 179, numerical aperture 0.22, tip diameter 2.5 mm; CeramOptec GmbH, Bonn, Germany) was used in which two branches were blocked and the other two guided the light to the phantom and the sensor separately. To calculate the light fluence on the surface of the phantom, the measured energy was divided by the illumination area on the surface of the phantom. A separation of 2.8 cm between the phantom and the transducer was used to locate the phantom at the focal area of the transducer. The optoacoustic signal was collected using a single-element transducer (Olympus, PZT, Waltham, MA, USA), with a focal length of 3.5 cm, a central frequency of 3.5 MHz, and a detector bandwidth of 90%. The signals were amplified using an AU-1291 amplifier (L-3 Narda-MITEQ, Hauppauge, NY, USA) and recorded using a data acquisition card (PCI-7340, National Instruments Corporate, USA). Each phantom was produced three times and each measurement was performed three times for each phantom.

To examine the nonlinear changes in optoacoustic pressure extracted from measured optoacoustic data of the phantom and the mouse kidney cross-section, the results of which are shown in Figs. 3–5, a small-animal multispectral optoacoustic tomography scanner (MSOT256-TF; iThera Medical, Germany) and imaging setup shown in Fig. 6b were used. This imaging system has been described in detail elsewhere^24^. A tunable OPO laser (InnoLas Laser) illuminated a water-filled field of view with a diameter of 4.05 cm. The phantom was uniformly illuminated from five side by guiding the light with a 10-branch (five pairs) fiber bundle (WF 179, numerical aperture 0.22, tip diameter 2.5 mm; CeramOptec). Fluences of 1.5 ± 0.02 and 9 ± 0.1 mJ/cm^2^ were used to acquire data. The data were detected using a 256-element ultrasound transducer array with a central frequency of 5 MHz and a detector bandwidth of 90%. Signals were amplified and recorded using a data acquisition card^24^. The acquired data was reconstructed after pre-processing the data with a 200 kHz- 8 MHz band-pass filter in order to remove frequencies that are far away from the frequency response of the transducer array.

### Temperature Control

In both phantom and in vivo measurements, temperature control was meticulously maintained. Samples were placed in a water tank with precisely controlled temperature, and measurements were only conducted after the samples had stabilized for at least 30 minutes. For in vivo experiments, the mouse was kept under anesthesia to prevent physiological activation, ensuring stable body temperature. Measurements at low and high laser energies were performed rapidly and consecutively to minimize potential temperature changes.

## Supplementary information

Supplementary Information

## Data Availability

The raw optoacoustic imaging data from animals and phantoms are available from the corresponding author and the first author upon request.
