# Supplementary material for: Nonlinearity of optoacoustic signals and a new contrast mechanism for imaging
Source: Light Sci Appl. 2025 Mar 27;14:142. doi: 10.1038/s41377-025-01772-7 (PMC11950213; doi:10.1038/s41377-025-01772-7)
Supplement: Supplementary file 1 — Supplementary Information [file 41377_2025_1772_MOESM1_ESM.pdf]

Supplementary Information for

Nonlinearity of Optoacoustic signals and a new contrast mechanism for imaging

Jaber Malekzadeh-Najafabadi[1,2,3], Jaya Prakash[1,2,4], Daniel Razansky[3,5], Jorge Ripoll[6], Vipul Gujrati[1,2], and Vasilis Ntziachristos[1,2,7*]*

[1]Chair of Biological Imaging, Central Institute for Translational Cancer Research (TranslaTUM), School of Medicine and Health & School of Computation, Information and Technology, Technical University of Munich, Munich, Germany

[2]Institute of Biological and Medical Imaging, Bioengineering Center, Helmholtz Zentrum München, Neuherberg, Germany

[3]Current address: Institute for Biomedical Engineering, Department of Information Technology and Electrical Engineering, ETH Zurich, Zurich, Switzerland.

[4]Current address: Department of Instrumentation and Applied Physics, Indian Institute of Science, Bengaluru, India.

[5]Institute for Biomedical Engineering and Institute of Pharmacology and Toxicology, Faculty of Medicine, University of Zurich, Switzerland.

[6]Department of Bioingeneering and Aerospace Engineering, Universidad Carlos III de Madrid, Madrid, Spain.

[7]Munich Institute of Robotics and Machine Intelligence (MIRMI), Technical University of Munich, Munich, Germany

*Email: jmalekzadehn@ethz.ch

*Email: bioimaging.translatum@tum.de

This document contains:

Supplementary Note 1: Theory

Supplementary Note 2: Absorption coefficient as a function of temperature

Supplementary Note 3: Grueneisen parameter as a function of temperature

Supplementary Note 4: Quantitative estimate of the magnitude of nonlinear variations in optoacoustic pressure

Supplementary Note 5: Two-Photon absorption (TPA)

Supplementary Note 6: Reproducibility of $\chi_{\mathrm{th}}^{(3)}$ Measurements

Supplementary Note 1: Theory

In order to study the origins of optoacoustic non-linearity, we calculate the generated temperature and pressure from an illuminated sample by investigating the effects of an electric field on a theoretical absorptive dielectric slab in a parallel plate capacitor (Figure S1). The dielectric slab has a relative permittivity of ε, a refractive index of $n = \sqrt{\varepsilon' + i\varepsilon"}$ and an absorption coefficient of $\mu_{\mathrm{a}} = \varepsilon"k$ (assuming the material is non-magnetic), $\varepsilon"$ is the imaginary part of the permittivity and k is the wavenumber. The electric field strength between the parallel-plate capacitor is *E*. When the dielectric slab is located between the capacitor plates, the potential energy per unit volume of the dielectric slab is given as,

$$u = \frac{1}{2}\varepsilon_0\varepsilon' E^2 \tag{S1}$$

where $\boldsymbol{\varepsilon_0}$ is vacuum permittivity. The heat generated per unit volume in the dielectric slab is,

$$\Delta Q = \mu_a \int I dt \tag{S2}$$

where $I = \varepsilon_0 ncE^2$ ($\mathrm{Js^{-1}m^{-2}}$) is the field intensity, with c representing the speed of light in a vacuum [1]. In the consideration of thermal effects, energy is the relevant quantity for pulsed lasers [1].

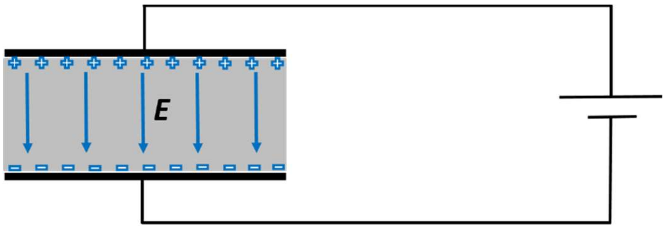

Figure S1. A dielectric slab located between parallel-plate capacitors. *E* is the electric field strength between the plates.

The second law of thermodynamics reads,

$$T\Delta s = \Delta QV \tag{S3}$$

where s is entropy ($\mathrm{J.K^{-1}}$), T is temperature (K) and V is volume (m^3). The change in the entropy is given by $\Delta s = \left(\frac{\partial s}{\partial T}\right)_p \Delta T$, where the change in entropy versus temperature at constant pressure (p) can be obtained using Maxwell relation,

$$\left(\frac{\partial s}{\partial T}\right)_p = \frac{MC_p}{T} \tag{S4}$$

where C_p represents specific heat capacity ($\mathrm{J.K^{-1}}.Kg^{-1}$) and M indicates the mass (Kg). Now the change in the temperature of the dielectric slab induced by the generated heat per unit of volume (Q) is given by (using Eq. (S3) and (S4)),

$$\Delta T = \frac{\mu_a \varphi}{\rho C_p} \tag{S5}$$

Where ρ indicates the mass density (Kgm^{-3}), $\varphi = \int I(t)\mathrm{d}t$ is the light fluence ($\mathrm{Jm^{-2}}$). The assumption that fluence (φ) inside the tissue is equivalent to $\int I(t)\mathrm{d}t$ in the parallel-plate capacitor model is based on the fact that the energy deposition in both contexts is related to time-integrated intensity. In this model, I is defined as $I = \varepsilon_0 ncE^2$, where E is the electric field strength. Thus, the integral $\int I(t)dt$ represents the total energy deposited, which, in optical terms, is defined as fluence. Eq. (S5) assumes adiabatic conditions, neglecting heat transport, which is a reasonable assumption on the timescale of photoacoustic signal generation.

The measured optoacoustic signal is the generated initial pressure rise inside the material that is propagated and then detected by the transducer. To find the main source of the nonlinear variations in optoacoustic signals, we should obtain a general form of the pressure equation, which includes all possible nonlinear parameters. We first calculate the pressure generated by the changes in the temperature. The change in pressure as a function of temperature at constant volume (V) can be written as,

$$\Delta p = \left(\frac{\partial p}{\partial T}\right)_V \Delta T \tag{S6}$$

Using Maxwell relations, we can also represent the change in pressure versus temperature at constant volume in terms of the thermal expansion coefficient,

$$\left(\frac{\partial p}{\partial T}\right)_V = \beta \rho v^2 \tag{S7}$$

where v is speed of sound (ms^{-1}), and β is the thermal expansion at constant pressure (K^{-1}). Now the pressure, thermally excited by the transferred heat, can be estimated by substituting Eq. (S5) and (S7) into Eq. (S6),

$$\Delta p = \frac{\beta v^2}{C_p} \mu_a \varphi \tag{S8}$$

By introducing the Grueneisen parameter ($\Gamma = \frac{\beta v^2}{C_p}$) and $\Delta p = p_{\mathrm{th}}$ as the thermally excited pressure into Eq. (S8), we obtain,

$$p_{th} = \Gamma \mu_a \varphi \tag{S9}$$

Eq. (S9) represents the optoacoustic pressure generated by the heat (ΔQ). Next, we investigate the other possible variations of the pressure induced by light inside the dielectric slab. We investigated the variation in the field energy density (Eq. (S1)) of the dielectric slab (Figure S1) due to the change in the permittivity, which is a function of temperature and pressure [1]. The potential energy density (Eq. (S1)) changes by the amount,

$$\Delta u = \frac{1}{2} \varepsilon_0 \Delta \varepsilon_{\mathrm{th}} E^2 \tag{S10}$$

where $\Delta\varepsilon_{\mathrm{th}}$ is the variation in the real part of permittivity due to change in temperature. Assuming that no external energy is added, according to the first law of thermodynamics at constant temperature, the change in energy (Eq. (S10)) equals the work performed on the system (dielectric slab),

$$\frac{\Delta w}{V} = \frac{\Delta pV}{V} = \Delta u \tag{S11}$$

where Δw $(Kg.m^2.s^{-2})$ is the change in work at constant volume. By introducing Eq. (S10) for the energy density into Eq. (S11), and using $I = \varepsilon_0 ncE^2$, the change in pressure (Δp) becomes,

$$\Delta p_{th} = \frac{\Delta\varepsilon_{th}}{2nc} I \tag{S12}$$

Δp_{th} is the change in optoacoustic pressure due to the thermally excited change in the permittivity $(\Delta\varepsilon_{\mathrm{th}})$. Next, we derive the acoustic wave equation for optoacoustic pressure (p_{th}) and the change in pressure (Δp_{th}) in order to investigate the nonlinear variations in the detected optoacoustic signal. Therefore, the dielectric slab (Figure S1) experiences a total pressure,

$$p_{\mathrm{tot}} = p_{\mathrm{th}} + \Delta p_{\mathrm{th}} = \Gamma\mu_a\varphi + \frac{\Delta\varepsilon_{\mathrm{th}}}{2nc} I \tag{S13}$$

By replacing φ with $\int I(t)dt$, we see that Δp_{th} is proportional to the derivative of thermal pressure,

$$\Delta p_{\mathrm{th}} \propto \frac{\partial p_{\mathrm{th}}}{\partial \mathrm{t}} \tag{S14}$$

To investigate the effects of the total pressure (p_{tot}) in optoacoustic imaging, we derived an acoustic pressure wave equation by using the Navier-Stokes and continuity equations [2-6],

$$\rho\left[\frac{\partial\vec{v}}{\partial t} + (\vec{v}\cdot\nabla)\vec{v}\right] = -\nabla p + F + \eta_s\nabla^2\vec{v} + (\eta_b + \frac{1}{3}\eta_s)\nabla(\nabla.\vec{v}) \tag{S15}$$

$$\frac{\partial\rho}{\partial t} + \nabla(\rho.\vec{v}) = 0 \tag{S16}$$

where $\vec{v}$ is velocity vector, η_s and η_b are shear and bulk viscosity, respectively, and *F* represents the force per unit volume, which is given by,

$$F = -\nabla p_{\mathrm{tot}} = -\Gamma\mu_a\nabla\varphi - \frac{\Delta\varepsilon_{\mathrm{th}}}{2nc}\nabla I \tag{S17}$$

By neglecting the convective acceleration term ($(\vec{v}\cdot\nabla)\vec{v}$) and the viscosity effects in Eq. (S15) and using Eq. (S16) and (S17), we find

$$\nabla^2 p(r,t) - \frac{1}{v^2}\frac{\partial^2}{\partial t^2}p(r,t) = -\frac{\Gamma\mu_a}{v^2}\frac{\partial}{\partial \mathrm{t}}\mathrm{I}(r,t) - \frac{\Delta\varepsilon_{th}}{2ncv^2}\frac{\partial^2}{\partial \mathrm{t}^2}\mathrm{I}(r,t) \tag{S18}$$

where the spatial derivatives were replaced by time derivatives by using $r = vt$ and $\nabla^2\varphi = \frac{1}{v^2}\frac{\partial \mathrm{I}}{\partial \mathrm{t}}$, where $\varphi = \int I(t)dt$. The change in the dielectric constant can be related to the third-order nonlinear susceptibility [1],

$$\Delta\varepsilon_{th} = \frac{\chi_{\mathrm{th}}^{(3)} I}{n\varepsilon_0 c} \tag{S19}$$

where $\chi_{\mathrm{th}}^{(3)}$ is the third-order nonlinear susceptibility due to thermal effects, which has a typical value of approximately $10^{-12}\ m^2V^{-2}$ [1]. In order to better understand the nonlinear variations contributing to the measured optoacoustic pressure, we solved the general form of the pressure wave equation (Eq. (S18)) for a symmetric cylindrical coordinate system along the z-axis. The changes in light intensity along the z-axis can be approximated as an exponential decay function with no changes in the radial direction,

$$I(z,t) = I_0 I_z I_t = I_0 \exp\left(-\mu_{\mathrm{eff}}\,\mathrm{z} - 4\frac{\mathrm{t}^2}{\mathrm{t}_0^2}\right) \tag{S20}$$

where I_0 represents the initial field intensity, $I_z = exp\,(-\mu_{\mathrm{eff}} z)$ and $I_t = exp\,(-4\frac{t^2}{t_0^2})$. The pressure wave ($p$) generated by the laser intensity (I) is given by Eq. (S18). The general solution of the pressure equation wave can be obtained by taking the Fourier transform of the both sides of Eq. (S18),

$$\nabla^2\tilde{p}(z,\omega) + k^2\tilde{p}(z,\omega) = S(z,\omega) \tag{S21}$$

where $\tilde{p}$ is the Fourier transform of p. $\mathrm{S}(\mathrm{z},\omega)$ is the source term,

$$S(z,\omega) = I(r,\omega)(-\frac{\Gamma\mu_a}{v^2}i\omega + \frac{\Delta\varepsilon_{th}}{2nc}k^2) \tag{S22}$$

Where $k = \frac{\omega}{v}$ and $I(r,\omega)$ is Fourier transform of Eq. (S20). By substituting $I(r,\omega) = I_0 I_\omega I_z$, $A = \frac{\Gamma\mu_a}{v^2}\omega$, and $B = \frac{\Delta\varepsilon_{th}}{2nc}k^2$ into Eq. (S22),

$$S(z,\omega) = I_0 I_\omega I_z(-iA + B) \tag{S23}$$

where I_ω is the Fourier transform of I_t. Eq. (S21) can be solved in the z-direction using Green's function in cylindrical coordinates. The boundary condition for the wave equation, which describes a wave traveling in both directions along the z-axis, is $z \in (-\infty, \infty)$. Eq. (S21) is a Sturm-Liouville problem of the third kind (SLP3) due to infinite boundary conditions, wherein the solution is given by,

$$\tilde{p}(z',\omega) = \int_{-\infty}^{+\infty} S(z,\omega) g(z,z') dz \tag{S24}$$

where $g(z,z')$ is Green's function (one dimension),

$$g(z,z') = \frac{e^{-ik|z-z'|}}{2ik} \tag{S25}$$

Therefore, the solution can be written:

$$\tilde{p}(z',\omega) = \frac{I_0 I_\omega}{2k}[(-A - iB)I_1']e^{-ikz'} + \frac{I_0 I_\omega}{2k}[(-A - iB)I_1]e^{ikz'} \tag{S26}$$

where $I_1' = \int_{-\infty}^{z'} I_z exp(ikz)\, dz$ and $I_1 = \int_{z'}^{+\infty} I_z exp(-ikz)\, dz$. Then, $\tilde{p}(z,\omega)$ can be rewritten by changing z' to z:

$$\tilde{p}(z,\omega) = \tilde{p}^+(z,\omega) + \tilde{p}^-(z,\omega) \tag{S27}$$

$$\tilde{p}^+(z,\omega) = -\frac{I_0 I_\omega I_1'}{2k}(A + iB) exp\,(-ikz) \tag{S28}$$

$$\tilde{p}^{-}(z,\omega) = -\frac{I_0 I_\omega I_1}{2k}(A + iB)exp\,(ikz) \tag{S29}$$

In Eq. (S27), the first term $\tilde{p}^{+}(z,\omega)$ is a wave traveling in the positive z-direction and the second term $\tilde{p}^{-}(z,\omega)$ is a wave traveling in the negative direction. If an acoustic detector (transducer) is placed at position $z < 0$, the detected acoustic signal would be $\tilde{p}^{-}(z,\omega)$, which includes both thermal optoacoustic pressure and nonlinear variations in optoacoustic pressure:

$$\tilde{p}_{\mathrm{th}}^{-}(z,\omega) = M_1 I_0 \tag{S30}$$

$$\Delta\tilde{p}_{\mathrm{th}}^{-}(z,\omega) = M_2 I_0^2 \omega \tag{S31}$$

where, $\tilde{p}_{\mathrm{th}}^{-}$ and $\Delta\tilde{p}_{th}^{-}$ are thermal pressure and the variations in optoacoustic pressure, respectively; $M_1 = -\frac{I_\omega I_1}{2v}\Gamma\mu_a exp\,(ikz)$ and $M_2 = -i\frac{I_\omega I_1}{2v}\frac{\chi_{th}^{(3)}}{2\varepsilon_0(nc)^2}exp\,(ikz)$. For simplicity, we considered $\Delta\varepsilon_{\mathrm{th}}$ as a function of I_0 instead of I. By approximating $\varphi \approx I\Delta t$ and using Eq. (S13) and (S19), the calculated initial pressure rise from Eq. (S18) can be written as,

$$p_{\mathrm{tot}} = p_{\mathrm{th}} + \Delta p_{th} = \Gamma\mu_a \Delta \mathrm{t}\,\mathrm{I} + \frac{\chi_{th}^{(3)}}{2\varepsilon_0(nc)^2}I^2 \tag{S32}$$

where Δt is the laser pulse duration. Eq. (S32) shows that p_{th} and Δp_{th} behave linearly and nonlinearly with light energy, respectively. Optoacoustic pressure includes both p_{th} and Δp_{th}, which should be unmixed (section 3.2). The nonlinear behavior of Δp_{th} allows it to be extracted from the measured total pressure (p_{tot}). Since the thermal pressure (p_{th}) is linear, the change in pressure (Δp_{th}) can be obtained by subtracting two normalized values of total optoacoustic pressure (p_{tot}) measured at two different light intensities. By using Eq. (S32), we find that

$$\Delta\tilde{p}_{\mathrm{tot}} = \frac{p_{\mathrm{tot2}}}{I_2} - \frac{p_{\mathrm{tot1}}}{I_1} = \frac{\chi_{\mathrm{th}}^{(3)}}{2\varepsilon_0(nc)^2}\Delta I \tag{S33}$$

where p_{toti} is the total optoacoustic pressure at fluence I_i and ΔI is $I_2 - I_1$. By comparing with Eq. (S32), we find that $\Delta\tilde{p}_{\mathrm{tot}}$ (Eq. (S33)) equals the normalized value of Δp_{th} for a given change in light intensity, ΔI. Then therefore obtain,

$$\Delta\tilde{p}_{\mathrm{tot}} \propto \Delta\tilde{p}_{\mathrm{th}} = \frac{\Delta p_{\mathrm{th}}}{I} \tag{S34}$$

where $\Delta\tilde{p}_{\mathrm{th}}$ is the normalized value of Δp_{th}.

Supplementary Note 2: Absorption coefficient as a function of temperature

In order to study nonlinear variations in optoacoustic pressure due to absorption coefficient, we calculate the wave equation by considering the absorption coefficient μ_a as a function of temperature. The absorption coefficient can be considered as a function of temperature as,

$$\mu_a \approx (\mu_a)_{\mathrm{T}=T_0} + \left.\frac{\partial \mu_a}{\partial T}\right|_{T=T_0} \Delta T \tag{S35}$$

where T_0 is equilibrium temperature of the sample and ΔT is the variation of temperature due to absorption of light that can be calculated from Eq. (S5). By using Eq. (S35), temperature can be calculated,

$$\Delta T \approx (\mu_a + \frac{\partial \mu_a}{\partial T}\Delta T)\frac{\varphi}{\rho C_p} \tag{S36}$$

Rearranging terms, the changes in the temperature can be calculated as,

$$\Delta T \approx \frac{\frac{\mu_a \varphi}{\rho C_p}}{1 - \frac{\partial \mu_a}{\partial T}\frac{\varphi}{\rho C_p}} \tag{S37}$$

By considering $\frac{\partial \mu_a}{\partial T}\frac{\varphi}{\rho C_p} \ll 1$, we can approximate Eq. (S37) to,

$$\Delta T \approx \mu_a \frac{\int I\,dt}{\rho C_p}\left(1 + \frac{\partial \mu_a}{\partial T}\frac{\int I\,dt}{\rho C_p}\right) = \frac{\mu_a}{\rho C_p}\int I\,dt + \frac{\mu_a}{\left(\rho C_p\right)^2}\frac{\partial \mu_a}{\partial T}\left(\int I\,dt\right)^2 \tag{S38}$$

By considering Temperature as the source of Eq. (S18) and neglecting Δp_{th}, we find that,

$$\nabla^2 p(r,t) - \frac{1}{v^2}\frac{\partial^2}{\partial t^2}p(r,t) = -\beta\rho\frac{\partial^2}{\partial \mathrm{t}^2}\Delta T(r,t) \tag{S39}$$

By substituting Eq. (S38) into Eq. (S39),

$$\nabla^2 p(r,t) - \frac{1}{v^2}\frac{\partial^2}{\partial t^2}p(r,t) = -\frac{\beta\mu_a}{C_p}\frac{\partial \mathrm{I}}{\partial \mathrm{t}} - \frac{2\beta\mu_a}{\rho\left(C_p\right)^2}\frac{\partial \mu_a}{\partial T}\frac{\partial \mathrm{I}}{\partial \mathrm{t}}\int I\,dt - \frac{2\beta\mu_a}{\rho\left(C_p\right)^2}\frac{\partial \mu_a}{\partial T}I^2 \tag{S40}$$

The last term $(\frac{2\beta\mu_a}{\rho\left(C_p\right)^2}\frac{\partial \mu_a}{\partial T}I^2)$ is generating a standing wave and can be removed from our calculations, giving,

$$\nabla^2 p(r,t) - \frac{1}{v^2}\frac{\partial^2}{\partial t^2}p(r,t) = -\frac{\beta\mu_a}{C_p}(1 + \frac{2}{\rho C_p}\frac{\partial \mu_a}{\partial T}\varphi)\frac{\partial \mathrm{I}}{\partial \mathrm{t}} \tag{S41}$$

Under standard conditions for our phantom measurements ($\varphi = 10\ mJcm^{-2}$, $\rho = 1000\ kgm^{-3}$, and $C_p = 4.18\ \mathrm{J(gK)}^{-1}$ for water at room temperature), the second term in the right side of Eq. (S41) is $\frac{2}{\rho C_p}\frac{\partial \mu_a}{\partial T}\varphi \approx 5 \times 10^{-5}\frac{\partial \mu_a}{\partial T}$. By considering $\frac{\partial \mu_a}{\partial T} < 1$, the second term in the right side of Eq. (S41) ($\frac{2}{\rho C_p}\frac{\partial \mu_a}{\partial T}\varphi \ll 1$) can be neglected,

$$\nabla^2 p(r,t) - \frac{1}{v^2}\frac{\partial^2}{\partial t^2}p(r,t) = -\frac{\beta \mu_a}{C_p}\frac{\partial \mathrm{I}}{\partial \mathrm{t}}(r,t) \tag{S42}$$

This result therefore proves that if the absorption coefficient is a function of temperature, it would not cause nonlinearity in the optoacoustic pressure. Moreover, the light induced temperature given the fluences used in our measurements is not sufficient to change absorption coefficient.

Supplementary Note 3: Grueneisen parameter as a function of temperature

To investigate the effects of temperature on optoacoustic nonlinearity, we must calculate optoacoustic pressure as a function of temperature. By considering Grueneisen parameter as a nonlinear function of temperature, we can write

$$\Gamma \approx \Gamma|_{T=T_0} + \left.\frac{\partial \Gamma}{\partial T}\right|_{T=T_0} \Delta T \qquad \text{(S43)}$$

where T_0 is equilibrium temperature of the sample and ΔT is the variation of temperature due to absorption of light. By substituting Eq. (S43) and Eq. (S5) ($\Delta \mathrm{T} = \frac{\mu_a \varphi}{\rho C_p}$,) into Eq. (S9) ($p_{\mathrm{th}} = \Gamma \mu_a \varphi$) and using $\Gamma = \frac{\beta c^2}{C_p}$, we find that

$$p = \Gamma \mu_a \varphi (1 + \frac{1}{\rho \beta c^2} \frac{\partial \Gamma}{\partial T} \mu_a \varphi) \qquad \text{(S44)}$$

The second term in Eq. (S44) represents nonlinearity due to light fluence (temperature). If we assume the second term is negligible, temperature rise is unlikely to explain the optoacoustic nonlinearity. By considering $\frac{\partial \Gamma}{\partial T} = 52 \times 10^{-4}\ (K^{-1})$ for water at room temperature [7] and under standard conditions for our phantom measurements ($\varphi = 10\ mJcm^{-2}$, $c\ = 1480\ \mathrm{m}s^{-1}$, $\rho = 1000\ \mathrm{kgm}^{-3}$, and $\beta = 210 \times 10^{-6}\ K^{-1}$ for water at room temperature, and $\mu_a = 0.1\ cm^{-1}$), we have $\frac{1}{\rho \beta c^2} \frac{\partial \Gamma}{\partial T} \mu_a \varphi \approx 1.13 \times 10^{-5}$. Consequently, the second term in Eq. (S44) inside the parentheses is negligible ($\frac{1}{\rho \beta\ ^2} \frac{\partial \Gamma}{\partial T} \mu_a \varphi \ll 1$) and optoacoustic pressure is $p = \Gamma \mu_a \varphi$, which is a linear function of fluence (temperature). However, the second term in Eq. (S44) has a singularity at $\beta = 0$. Therefore, the second term can have a significant value near the singularity in which case the optoacoustic pressure is nonlinear as a function of fluence. For example, the thermal expansion of water becomes zero at 4 $°C$ [8]. Therefore, the optoacoustic pressure might become nonlinear at low temperatures (close to 4 $°C$) for phantoms made

of water. We performed two experiments to investigate this hypothesis with a homogeneous agar cube phantom.

We initially performed an experiment to investigate the influence of temperature of the media on the optoacoustic pressure with a homogeneous agar cube with uniform absorption coefficient $\mu_a = 0.1 \pm .02\, cm^{-1}$ and reduced scattering coefficient $\mu_s{}' = 4 \pm 1\, cm^{-1}$ (See Methods section) which was illuminated at 10$\pm$.4 $mJcm^{-2}$ (800 nm) in transmission mode (Figure 5(a)). The temperature of the phantom was controlled, and the optoacoustic signal was acquired at five different temperatures 0.5$\pm$.2, 2.5$\pm$.2, 6.0$\pm$.2, 10.80$\pm$.2, and 17.0$\pm$.2 degree Celsius. Figure S2(a) shows the maximum value of the optoacoustic signal as a function of the temperature of the media. According to Figure S2a, optoacoustic pressure is a linear function of temperature at a constant fluence.

Moreover, we performed another experiment to explore the nonlinear behavior of the optoacoustic signal as a function of fluence at different temperatures by controlling the temperature of the media.

In this experiment, we created a phantom of intralipid, pure water, ink, and agar, such that the reduced scattering coefficient was 4$\pm$1 cm^{-1} and absorption coefficient was $\mu_a = 0.1 \pm .02\ \mathrm{cm}^{-1}$ (See Methods section). Then the phantom was illuminated with different fluences at different temperatures. Figure S2b shows the maximum of the optoacoustic signal as a function of light fluence at different temperatures. Figure S2b-c show that optoacoustic signals decrease with reducing temperature and become nonlinear at lower temperatures. Therefore, this observation is consistent with our theoretical results (Eq. (S44)), suggesting that optoacoustic pressure is nonlinear near the singularity (4 $°C$ for water). In addition, since the phantom is made of both water and ink, the thermal expansion is not zero exactly at 4 $°C$. Consequently, the observed nonlinearity in our phantom measurements at room temperature cannot be explained by the laser-induced variations in temperature.

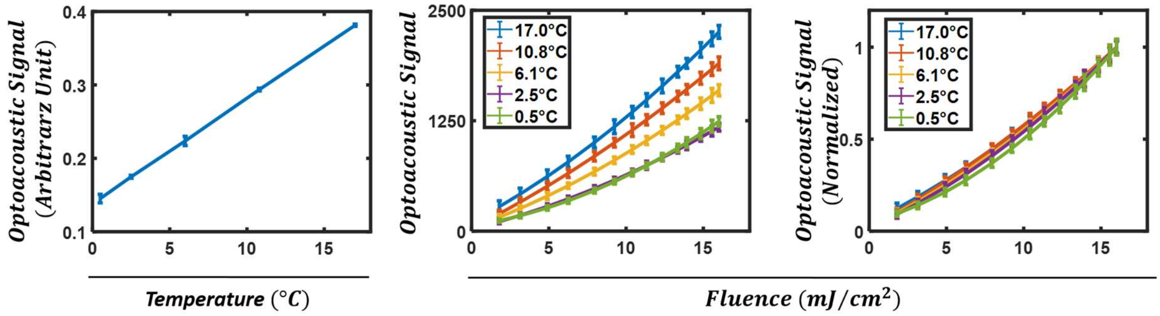

Figure S2. Exploring influence of temperature on optoacoustic pressure. A homogeneous agar cube with uniform absorption coefficient $\mu_a = 0.1 \pm .02\ cm^{-1}$ and reduced scattering coefficient $\mu_s{}' = 4\ \pm 1cm^{-1}$ was illuminated at $10\ \pm .4\ mJcm^{-2}$ (800 nm) as a function of temperature. (a) Normalized maximum value of the acquired optoacoustic signal at light fluence $10 \pm .4\ mJcm^{-2}$ as a function of temperature. (b) The maximum of the detected signal over light fluence at five different temperatures. (c) Normalization of the data in panel (b).

Supplementary Note 4: Quantitative estimate of the magnitude of nonlinear variations in optoacoustic pressure

By using Eq. (S32) ($\Delta p_{th} = \frac{\chi_{th}^{(3)}}{2\varepsilon_0 (nc)^2} I^2$) and under standard conditions for our phantom measurements ($I \approx \frac{\varphi}{\Delta t} = \frac{10mJcm^{-2}}{10^{-8}s} = 10^{10} (Wm^{-2})$, $\varepsilon_0 = 8.85 \times 10^{-12}\ (Fm^{-1})$, $n = 1.33$, and $c = 3 \times 10^{8} (ms^{-1})$, $\chi_{\mathrm{th}}^{(3)} = 10^{-12}\ (m^2 V^{-2})$([1], Table 4.1.1, page 211), the light-induced change in pressure is $\Delta p_{\mathrm{th}} = 35.5(\mathrm{Jm}^{-3})$, which is generally too large compared to the change in pressure due to nonlinear Gruneisen parameter which is calculated from Eq. (S44), $\Delta p_{\Gamma} = \frac{\Gamma}{\rho \beta c^2} \frac{\partial \Gamma}{\partial T} (\mu_a \varphi)^2 \approx 5.3 \times 10^{-4} (Jm^{-3})$, ($\Gamma = \frac{\beta v^2}{\mathrm{C_p}} \approx 0.11$ for water). According to our calculations, we have $\Delta p_{\mathrm{th}} \gg \Delta p_{\Gamma}$. Therefore, the nonlinearity arising from the Gruneisen parameter is insufficient to explain the observed nonlinearity in our measurements.

Supplementary Note 5: Two-Photon absorption (TPA)

We can model the total absorption process using the equation that accounts for both single-photon and two-photon absorption [1],

$$\alpha = \alpha_0 + \beta I \qquad \text{(S45)}$$

Where α_0 is the single-photon absorption coefficient (cm^{-1}), also denoted as μ_a, β is the two-photon absorption coefficient (cmW^{-1}), and I is the laser intensity (Wcm^{-2}). When the standard conditions for tissue mimicking phantom measurements are inserted into Eq. **Error! Reference source not found.** (with $\alpha_0 = 0.1\, cm^{-1}$, $\beta \approx 10^{-10} cmW^{-1}$ for water, $I \approx \frac{\varphi}{\Delta t} = \frac{10\mathrm{mJcm^{-2}}}{10^{-8}s} = 10^6\, Wcm^{-2}$). Substituting these values into Eq. (S45) results in $\alpha = 0.1\, cm^{-1} + 10^{-4}\, cm^{-1}$.This shows that the contribution of two-photon absorption (βI) is very small, adding only $10^{-4}\, cm^{-1}$ to the total absorption. The single-photon absorption dominates, contributing $0.1\, cm^{-1}$, while the two-photon absorption contribution is negligible by comparison.

Given these results, it is clear that the intensity is too low to induce significant nonlinear effects through TPA. Typically, **picosecond or femtosecond lasers** are used for two-photon absorption because they produce much higher peak intensities, in the $GWcm^{-2}$ range, which are required to drive TPA effectively. Therefore, in your case, the nonlinear effects are primarily governed by single-photon absorption.

Supplementary Note 6: Reproducibility of $\chi_{th}^{(3)}$ Measurements

We repeated the optoacoustic measurements three times at two light fluences:

1. $\varphi_{\min} = 2.00 \pm 0.02$ and $\varphi_{\max} = 8.96 \pm 0.12\ \text{mJcm}^{-2}$,
2. $\varphi_{\min} = 2.22 \pm 0.02$ and $\varphi_{\max} = 8.70 \pm 0.12\ \text{mJcm}^{-2}$,
3. $\varphi_{\min} = 1.98 \pm 0.02$ and $\varphi_{\max} = 8.71 \pm 0.11\ \text{mJcm}^{-2}$.

The reconstructed images (Figure S3) show results from applying the modified model-based algorithm (Eq. (14)). The average and variability of the measurements across different regions of interest (ROIs) were evaluated to assess consistency.

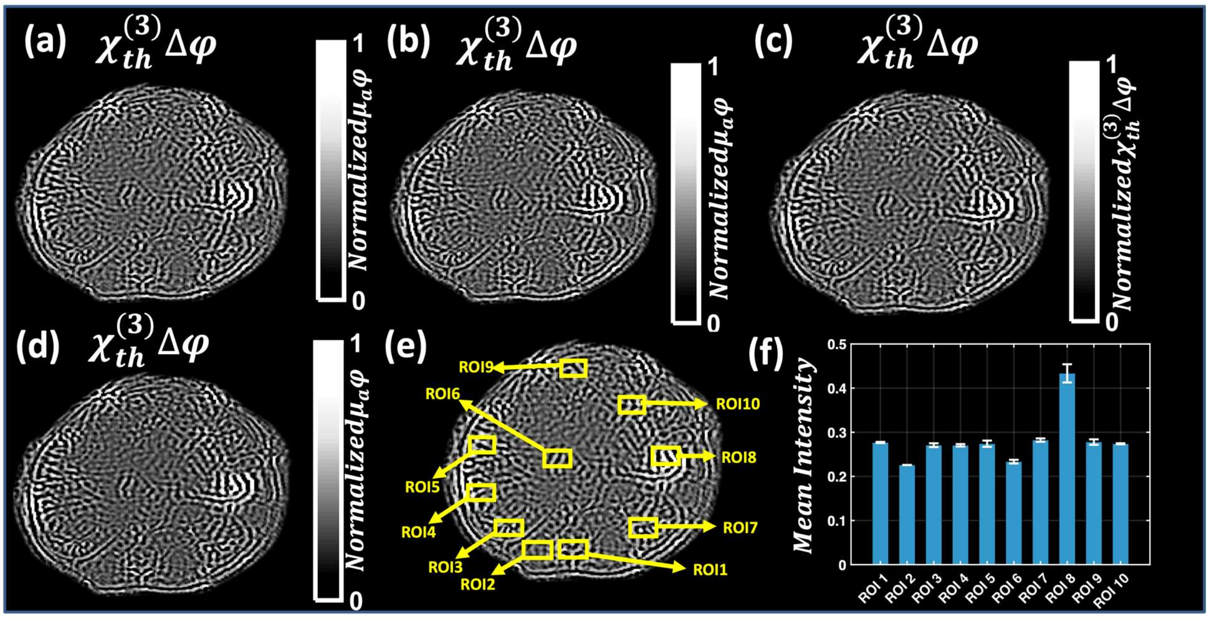

Figure S3. $\chi_{\text{th}}^{(3)}$ imaging results from three repeated measurements. (a-c) $\chi_{\text{th}}^{(3)}$ images reconstructed using the modified algorithm from three different sets of fluence data. (d) The average image of the three measurements at panels (a-c). (e) Ten selected ROIs on the mean image. (f) Mean intensity values and standard deviation of the three measurements in each ROI from panels (a-c).

References

1. Boyd, R.W., *Nonlinear Optics*. Third ed. 2008: Elsevier.
2. Hutchins, D. and A.C. Tam, *Pulsed photoacoustic materials characterization.* IEEE Trans Ultrason Ferroelectr Freq Control, 1986. **33**(5): p. 429-49.
3. H. M. Lai and K. Young, *Theory of the pulsed optoacoustic technique.* J. Acoust. Soc. Am. 72, 2000, 1982.
4. I. Brevik, *Experiments in phenomenological electrodynamics and the electromagnetic energy-momentum tensor.* Phys. Rep. , 1979. **52**: p. 133.
5. Lou, C.G. and D. Xing, *Photoacoustic measurement of liquid viscosity.* Applied Physics Letters, 2010. **96**(21).
6. Cao, Y.N. and G.J. Diebold, *Effects of heat conduction and viscosity on photoacoustic waves from droplets.* Optical Engineering, 1997. **36**(2): p. 417-422.
7. Wang, W. and A. Mandelis, *Microwave-heating-coupled photoacoustic radar for tissue diagnostic imaging.* Journal of Biomedical Optics, 2016. **21**(6).
8. Prakash, J., et al., *Short-wavelength optoacoustic spectroscopy based on water muting.* Proceedings of the National Academy of Sciences, 2020. **117**(8): p. 4007-4014.
